# Physical Health Problems and Environmental Challenges Influence Balancing Behaviour in Laying Hens

**DOI:** 10.1371/journal.pone.0153477

**Published:** 2016-04-14

**Authors:** Stephanie LeBlanc, Bret Tobalske, Margaret Quinton, Dwight Springthorpe, Bill Szkotnicki, Hanno Wuerbel, Alexandra Harlander-Matauschek

**Affiliations:** 1 Department of Animal and Poultry Science, University of Guelph, Guelph, Ontario, Canada; 2 Division of Biological Sciences, University of Montana, Missoula, Montana, United States of America; 3 Department of Integrative Biology, University of California, Berkeley, California, United States of America; 4 Division of Animal Welfare, VPH Institute, University of Bern, Bern, Switzerland; Brown University, UNITED STATES

## Abstract

With rising public concern for animal welfare, many major food chains and restaurants are changing their policies, strictly buying their eggs from non-cage producers. However, with the additional space in these cage-free systems to perform natural behaviours and movements comes the risk of injury. We evaluated the ability to maintain balance in adult laying hens with health problems (footpad dermatitis, keel damage, poor wing feather cover; n = 15) using a series of environmental challenges and compared such abilities with those of healthy birds (n = 5). Environmental challenges consisted of visual and spatial constraints, created using a head mask, perch obstacles, and static and swaying perch states. We hypothesized that perch movement, environmental challenges, and diminished physical health would negatively impact perching performance demonstrated as balance (as measured by time spent on perch and by number of falls of the perch) and would require more exaggerated correctional movements. We measured perching stability whereby each bird underwent eight 30-second trials on a static and swaying perch: with and without disrupted vision (head mask), with and without space limitations (obstacles) and combinations thereof. Video recordings (600 Hz) and a three-axis accelerometer/gyroscope (100 Hz) were used to measure the number of jumps/falls, latencies to leave the perch, as well as magnitude and direction of both linear and rotational balance-correcting movements. Laying hens with and without physical health problems, in both challenged and unchallenged environments, managed to perch and remain off the ground. We attribute this capacity to our training of the birds. Environmental challenges and physical state had an effect on the use of accelerations and rotations to stabilize themselves on a perch. Birds with physical health problems performed a higher frequency of rotational corrections to keep the body centered over the perch, whereas, for both health categories, environmental challenges required more intense and variable movement corrections. Collectively, these results provide novel empirical support for the effectiveness of training, and highlight that overcrowding, visual constraints, and poor physical health all reduce perching performance.

## Introduction

With 5 billion laying hens (*Gallus gallus domesticus*) used worldwide for egg production [1], improvements in animal welfare have the potential to positively impact an enormous population of animals. One of the most challenging poultry issues is keel bone fractures, in which the breast bone is damaged. Epidemiological studies have showed that keel bone damage has a prevalence of 23%–96% [2–5]. These rates translate to billions of birds with bone fractures. Despite more than 70 years of research and efforts to alleviate this major problem [6–10] its causes are not fully understood and no practicable solution has yet emerged. In general, there are genetic, environmental, and nutritional factors associated with the onset of keel bone fractures [11], but their relative importance and interactions are unknown.

Falls cause ~95% of bone fractures among elderly humans [12]. To avoid falls and subsequent injuries, it is crucial that individuals have proper balance strategies [13]. Balance strategies allow humans to maintain postural stability by coordinating internal (generated during body movements) and external (gravity/interaction with the environment) forces applied on the body, which displace the center of mass [14]. Controlling the center of mass is accomplished by properly timed sensorimotor strategies [13, 15] such as the extension of arms in humans to improve postural stability [16]. The key sensory inputs involved in maintaining balance are the somatosensory (touch, temperature, nociception, proprioception), visual, and vestibular systems[17–19].The loss of any sensory capability, especially the loss of two or more, diminishes balance and subsequently increases the risk of falling [20]. After sensory input is transmitted and integrated in the brain, a response is executed by the musculo-skeletal system to maintain balance [15]. If any of these sensory input systems [20] and/or the musculoskeletal output [21–23] are impaired or if environmental challenges, such as inadequate lighting, unstable surface/furniture or obstructed walkways [24] are encountered, the body’s ability to maintain balance is compromised and the risk of falls and related bone fractures increases. As with bone fractures in humans [13, 25], a path toward mitigating keel bone fractures in birds is to understand challenges to postural stability and balance strategies.

Birds are highly adapted for life in the visual world, having eyes with sophisticated optical systems [26]. Vision is key source of information for birds, which they use to discern "patterns of optic flow" and to maintain balance [27–28]. Living organisms capable of vision react to these patterns, which are caused by the reflection of light from objects in the environment onto the retina of the eye during movement (e.g. swaying backwards to correct posture in response to visual stimuli subsequent to swaying forward towards an object)[27–28].

To control posture and motor output during perturbations, birds need an intact sense of equilibrium [29]. In birds, the vestibular organ in the inner ear functions during flight and a unique specialization in the lumbosacral region of the spinal cord functions as an equilibrium sense organ when walking on the ground or when sitting on perches [30].The control of motor output is a complex interaction of cognitive and sensorimotor functions[31]. A bird’s behavioural response following a sensory input, for example, while perching, being pushed by conspecifics in crowded conditions or being buffeted by high winds (in the wild), is to actively balance on the perch via contraction of the flexor muscles of the toes. The toes provide a saddle-like structure to balance the body over the central pad of the foot [32]. Somatosensory receptors in the skin, muscle, and joints are sensitive to stretch and pressure [19], which are applied to the skin of the foot pads during perching [33].

Another muscular response to balance disturbance in birds is wing flapping [34–35]. Potentially involving an aerodynamic force alongside the inertial torques produced, this stabilizes the center of mass, which in the domestic chicken is located anterior to the pelvic girdle and vertically aligned with the feet when standing [36–37]. The massive pectoralis major and supracoracoideus muscles, which originate from the keel bone [38], are the primary actuators for wing flapping during flight [39–40] and during balance attempts [41]. Wing loading, body weight relative to wing area, provides an index of the capacity for the wings to be useful during the balancing process. Research indicates that keel bone fractures in birds are painful [42] and thus, may result in reduced use of wings or ultimately in the degeneration or disuse atrophy of these muscles.

In humans, impaired vision, painful foot conditions, and dynamic challenges increase the risk of falls and subsequent injuries. Similar risk factors can be identified in loose (cage-free) housing systems for commercial laying hens. Low lighting is often used for reducing the incidence of damaging bird-to-bird pecking, but this may also limit the amount of visual information available for properly timed reaction to disturbances of balance [43]. Bird-to-bird pecking is widespread in laying hen systems and is the main cause for poor feather cover via damage to/ loss of feathers [44]. Feather damage may impact the structural cohesiveness of the feathers and lower the aerodynamic capacity of the wings [45–47], making them less efficient in helping to maintain balance. Wing porosity can be used to better understand the effect of poor wing feather cover on balancing ability. In addition, skin conditions such as footpad dermatitis, a well-known problem in laying hens [48], may alter the sensory input from cutaneous nerve endings located in the avian foot skin [41] as well as the motor output associated with balance perturbation on a perch.

In the present study, we measured responses to environmental challenges categorized as (a) crowded (dummy birds placed on either side of test bird) with disrupted vision (head mask placed over eyes), (b) crowded with vision, (c) non-crowded with disrupted vision and (d) non-crowded with vision. All four challenges were applied to both static (non-swaying perch) and swaying (dynamic perch) conditions, with the swaying perch designed to emulate the way tree branches might sway rhythmically in the wind. Since the interaction between physical abilities and exposure to environmental challenges appears to be important in mammals [20], we tested laying hens with physical health problems, including keel damage, foot pad dermatitis and poor wing feather cover under different environmental conditions. To measure postural responses to environmental challenges, we used multi-axial accelerometers/gyroscopes [49–51] coupled to data loggers.

We developed three main hypotheses for this study based on factors likely to diminish perching performance: (a) intense perch movements due to increased swaying, (b) environmental challenges due to reduced space and visual cues and (c) impaired physical health due to injury or disease. We hypothesized that each factor would increase the risk of falls, reduce perching time, and require more frequent and more intense correction movements.

## Materials and Methods

### Ethical Statement

This study was approved by the University of Guelph Animal Care Committee (Animal Utilization Protocol Number 2501) prior to testing.

### Birds and Management

Twenty heritage Shaver White Leghorn laying hens were obtained from the Arkell Research Station at the University of Guelph from a group of 50 hens and three roosters kept in a pen located inside a windowless room under 14:10 hours L:D lighting.

Birds were selected at 60 weeks of age for the presence of keel bone damage, foot pad dermatitis (grade III) [52], and poor wing feather cover (missing and/or broken feathers) [53] with the help of an experienced technician not involved in the present experiment. Using a palpation technique developed by Wilkins et al. [54], keel bone damage was assessed by running the index and thumb along the carina sterni (ventral part of the keel bone) to detect any discontinuities, callus formations, or deviations. We observed no acute inflammation manifested clinically by redness, heat, swelling, pain, and loss of function of the affected carina sterni. Hens with keel damage, foot pad dermatitis, and wing feather damage were selected in groups of five. Five physically healthy birds unaffected by any of these health conditions were selected as the control group.

Birds were transferred from the home pen to one of four floor pens (183L x 244W x 290H cm) and kept on wood shavings. The pens contained an elevated slatted platform (125L x 61W x 41H cm) holding three nest boxes (74L x 44W x 50H cm). A wooden perch measuring 5 cm in diameter and 152cm in length placed 41 cm from the ground, five nipple drinkers and a round feeder (38 cm diameter) were available with *ad libitum* access to a commercial standard layer-breeder ration (crumbles; ME (MJ/kg) = 11.7, CP = 17%) and water. Light was provided artificially from 0300h to 1720h with transitional periods of 20 minutes beginning at 0300h and 1700h. Light intensity in the room was 25 lux at bird-eye height. Custom backpacks (14.5 cm x 6 cm x 0.2 cm) were made from two plastic coated flexible wire straps attached by clothing rivets to two sheets of silicone and were fitted to the birds for identification purposes as described in Harlander-Matauschek et al. [55]. Each bird was assigned a number between 1 and 20, which was written on the silicone backpacks. The birds were habituated to the backpacks and housing environment for one week.

### Swaying Perch

The birds were tested for balance skills on a custom built, mechanically operated, swaying perch (square rod with rounded edges, 2.5 cm diameter, 88 cm long) located in a test arena (366 cm L x 488 cm W x 290 cm H). The perch was assembled within a wooden base structure comprised of a platform padded with foam (Interlocking play foam floor tiles, Canadian Tire ^®^, Guelph ON), two side walls and one back wall. A direct-current 120V motor (Model Y, Brevel Motors, Carlstadt, NJ, U.S.A.) controlled the perch through a v-belt pulley system (Innoda v-belt pulleys, The Innoda Group Ltd., Scarborough, ON, Canada). Different sized pulleys (15 cm and 20 cm diameters) allowed for different average perch speeds (training: 0.16 m/s; testing: 0.30 m/s) along the same excursion distance of 30 cm in one direction. All walls in the testing space within the bird’s field of vision were covered in white corrugated plastic sheets to provide a uniform environment. Three miniature LED indicator lights (white light emitting diode, LED, 0.5cm in diameter, ~40 lumens per Watt)located at the front edge of the platform and on the top beam of the perch apparatus, within view of the bird, signalled 2.5 seconds before the start of each test.

### Procedure

Each bird was tested under four environmental challenges: (a) crowded (dummy birds placed on either side of test birds) with disrupted vision (hood placed over eyes), (b) crowded with vision, (c) non-crowded with disrupted vision and (d) non-crowded with vision. All four challenges were applied to both static (non-swaying perch) and dynamic (swaying perch) states. A realistic crowding environment was simulated using two dummy chickens manufactured as non-toxic dog toys (Kooky Chicken, Petstages^™^) fastened to the perch 15 cm apart (one on either side of the hen) in accordance with perch space requirement in the European Union (EU Directive 1999/74/CEC). Disrupted vision was simulated by placing a lightweight, black cotton head mask on the birds, blocking all light (verified by human vision) and visual cues from the environment.

### Habituation and Training

The birds were habituated and trained for a period of four weeks prior to testing. Since the birds were to be exposed to various novel stimuli over the course of the experiment, they were systematically introduced to each of these gradually. Positive reinforcement in the form of food rewards (i.e. raisins and mealworms), was used to facilitate handling and use of the perch, transport crates, and testing apparatus. In the third week, each bird was handled for approximately five minutes for routine examinations of the keel bone, feather cover, and foot pads, and was rewarded with one raisin individually before being returned to its pen.

### Testing

The birds were tested in a randomized order across environmental challenges in dynamic and static conditions for 30 seconds for each condition. The accelerometer/gyroscope (see below) was enclosed in a plastic case (5.5 x 3.5 x 1.5 cm) lined with cotton to prevent the device from moving within the case, and was fastened to each bird via the silicone backpacks using Velcro^®^.

### Data Collection

Physical activity was quantified using a radio data logger (4.5 x 2 x 0.5 cm) weighing 6g (DAQpack; Corvus Scientific, Albany, CA, U.S.A.). The data logger included both a 3-axis accelerometer and 3-axis gyroscope (configured for +/- 8 g and +/- 1000 degrees/sec respectively; MPU6000; InvenSense, Sunnyvale, Ca, USA), which measured linear acceleration and angular velocities respectively. Acceleration and rotation rates about the x (forward/backward), y (left/right) and z (up/down) axes relative to the bird were sampled at 100 Hz for 30 seconds in all environmental factor treatments (S1 Fig). A computer equipped with a custom radio transceiver (DAQpack Base Station; Corvus Scientific, Albany, CA, USA) collected the logger’s data. Collected data in S1 Dataset logger data in Matlab format files and R code files was processed using Matlab^®^ (Mathworks Inc., Natick, MA, U.S.A.). The moving standard deviation of the readings (defined as activity counts) from the loggers was calculated for each of the axes (x, y and z). A peak-finding algorithm [56] counted the number of peaks (activity counts) per 30 second period that were above an empirically-determined inactivity threshold. Video footage (JVC GC-PX100, Full HD Everio Camcorder, JVC America Corp., NJ, U.S.A.; 600 fps) of the swaying perch tests was used to validate the inactivity threshold as well as to identify falls and jumps off the perch in S1 Video. The start of jumping was defined as a countermovement featuring bending of the legs, lowering of the breast, and drawing the neck in immediately prior to leaving the perch [57]. Falls were defined as leaving the perch without performing pre-jump, countermovement behaviour.

### Wing Measures

The birds were weighed to the nearest gram throughout the study using a digital electronic scale (Pennsylvania Scale, model 7500, Pennsylvania Scale Company, Lancaster, PA, U.S.A.). Each bird was weighed a total of three times (morning, noon, late afternoon) to account for the extra weight of eggs in the reproductive tract, as the birds were observed to lay irregularly throughout the day. The average of the three weights per bird (overall weight) was used to compute wing loading for each bird.

Images of the left and right wing for each bird were captured (JVC GC-PX100Full HD Everio Camcorder, JVC America Corp., NJ, U.S.A.) with the ventral side against a dark, uniform background and analysed using ImageJ software. Wing area (cm^2^) was calculated by tracing the outline of the wings. Body mass divided by the sum of the left and right wing areas was used to calculate wing loading [58]. Whole-wing porosity [46] was calculated using the actual wing area, defined by tracing the outline of the wing, and potential wing area defined by tracing the leading edge of the wing and the tips of the primary and secondary feathers, which corresponds to the image space that would be filled if the bird had no wing feather damage or feather loss.

### Statistical analysis

Maximum linear acceleration (highest corrective acceleration), standard deviation (variation) of acceleration and number of acceleration peaks (corrective acceleration events) as well as maximum angular velocity, angular standard deviations and number of peaks (corrective rotation events) in angular velocity along and about the three axes were measured. For each bird, these parameters were analyzed in accordance with the time the birds spent on the perch and the number of falls and/or jumps from the perch via a split plot design, in which the four physical health states (keel damage, foot damage, poor wing feather cover, healthy) were the main plot factor categorising each bird, and the four environmental challenges (mask, obstacle, and combinations thereof) and two perch states (swaying vs. static) were the subplot factors. All calculations were performed using PROC MIXED of the SAS System (Version 9.1.3, SAS Institute Inc., Cary, NC, U.S.A.).As described by Wang and Goonewardene [59], the standard way of handling three different movement dimensions with repeated measurements in space over the three axes (x, y, z) for each of the eight tests per bird is by using the model with the best-fitting variance/covariance structure among the three axes. The standard deviation and activity counts for each 30 second period were compared among treatment groups. All standard deviations and peak counts were square-root transformed prior to analysis to stabilize variances. The model included bodyweight, wing loading, and wing porosity as covariates; physical health problem, challenge (none, obstacle, mask, both obstacle and mask), perch state (swaying, static), axis (x, y, z) and all interactions among these as fixed effects; and laying hen within a physical health problem class and laying hen/test combination as random effects.

Differences among the means of the four environmental challenges and the means of the environmental challenge by state interaction were assessed using three orthogonal contrasts and their interactions with perch state: environmental challenge versus no environmental challenge, obstacle versus mask, and the average of obstacle and mask versus both obstacle and mask. A generalized linear mixed model was used to analyze the number of jumps/falls with the fixed factors physical health problem, challenge, state, wing porosity and bodyweight. Laying hen/physical health combination was the random effect. A binomial distribution best fit the data. Overall degrees of freedom were adjusted using the Satterthwaite method [60]. A logit link function and all analyses were performed using PROC GLIMMIX of the SAS System. The results are presented as least-square means data ± SE, if not otherwise stated.

## Results

### Falls, Jumps and Latencies

There was a significantly higher number of falls from the swaying perch (0.4 ± 0.08) than from the static perch (0.04 ± 0.02) (F_1,121_ = 31.62, *P* < 0.0001). Birds with higher body weights had a lower number of falls from the perch overall (F_1,14.26_ = 6.08, P < 0.03). There was a significant effect of environmental challenges on the number of jumps from the swaying perch (Obstacle (0.12 ± 0.03), Mask (0 ± 0), Obstacle + Mask (0 ± 0), Non-challenged (0.16 ± 0.42; F_3,121_ = 8.24, *P* < 0.0001); birds with obstructed vision did not ever jump from the swaying perch. In terms of latency, birds left the swaying perch significantly earlier than the static perch (19.11 ± 1.36 sec versus 28.53 ± 1.36 sec; (F_1,121_ = 40.25, *P* < 0.0001). Healthy birds and birds with keel damage remained longer on the static perch (30.6 ± 2.7 sec each) than birds with foot pad dermatitis (27.7 ± 2.7 sec) and feather damage (26.4 ± 2.7 sec; S2 Fig).There was no significant effect of physical health status on the latency (seconds) to leave the perch (static or swaying), but a rank order from the numerically shortest to the longest latency showed: foot pad dermatitis (22.6 ± 2.3 sec), feather damage (22.8 ± 2.3 sec), healthy birds (24 ± 2.3 sec) and birds with keel damage (26.1 ± 2.3 sec). However, there was a significant interaction between environmental challenges and physical health conditions on the latency to leave the perch (static or swaying) (F_9,121_ = 2.25, *P* < 0.0228; S2 Fig), which could be attributed to birds with foot (t_121_ = 2.50, *P* < 0.01) and feather damage (t_121_ = 1.98, *P* < 0.0499) leaving the perch earlier than healthy birds in the non-challenged condition. There was no significant difference in the time taken to leave the perch between birds with keel bone damage and healthy birds in the non-challenged condition.

### Angular velocity

There were significant main effects of perch state (F_1, 113_ = 143, *P*< 0.0001), physical health status (F_3, 74_ = 3.54, *P*< 0.02), and axis (F_2, 108_ = 82.6, *P*< 0.0001) on the number of peaks in angular velocity (S3A Fig). There were more high-intensity rotational movement corrections on the swaying perch (5.6 ± 0.2) than on the static perch (1.8 ± 0.2). There were more peak velocities for birds with physical health problems (4.0 ± 0.35) versus control (2.9 ± 0.3) birds (t_107_ = -3, *P* < 0.03; S3A Fig). Lastly, there were more peaks to regain balance along the mediolateral (y) axis (4.6 ± 0.2), followed by the craniocaudal (x) axis (3.78 ± 0.2), and dorsoventral (z) axis (2.8 ± 0.1). There was a significant interaction of perch state and axis (F_2, 108_ = 42, *P*< 0.0001) in for the number of peaks of angular velocity where differences between x and y axis on the static perch were lower [x- axis swaying (5.6 ± 0.2), static (1.87 ± 0.2); y-axis swaying (7.2 ± 0.38), static (2.0 ± 0.3); z-axis swaying (4.1 ± 0.18), static (1.54 ± 0.18)]. Birds with more porous wings performed more rotational body movements (F_1, 61_ = 4.46, *P*< 0.039). Birds with higher wing loading performed fewer rotational body movements (F_1, 61_ = 10.06, *P*< 0.0024).

Maximum intensity of angular velocity was varied significantly according to perch state (F_1, 86.4_ = 1874.28, *P*< 0.0001), environmental challenge (F_3, 86.4_ = 3.04, *P*< 0.0331), and axis of rotation (F_2, 115_ = 31.50, *P*< 0.0001; S3B Fig). The most intense corrective motions were performed on the swaying (983.7 ± 8.6 degrees s^-1^) versus the static perch (127 ± 18.3 degrees s^-1^) and in the non-environmental challenged (598.7 ± 20 degrees s^-1^) versus the challenged (540.9 ± 20 degrees s^-1^) condition (t_86.4_ = 2.53, *P* < 0.0251). The greatest intensities were exhibited about the cranio-caudal, x-axis (585. ± 12 degrees s^-1^) compared with the mediolateral y (561.8 ± 11 degrees s^-1^) and dorsoventral axes (z, 519.3 ± 10.3 degrees s^-1^). Healthy birds did not differ significantly from birds with physical health problems in the highest rotation rates performed to maintain balance.

For a given average angular velocity, the variance about the mean (angular standard deviation) exhibited differences among treatments. Birds exhibited less variation on the static perch than on the swaying perch (3.12 ± 0.3 degrees s^-1^ vs. 13.3 ± 0.4 degrees s^-1^; F_1, 86_ = 468.8, *P*< 0.0001) and more about the craniocaudal (x) axis (8.67 ± 0.3 degrees s^-1^) axis than the mediolateral (y) 8.28 ± 0.3 degrees s^-1^ and dorsoventral (z) 7.75 ± 0.27 degrees s^-1^ axes (F_2, 98_ = 81.7, *P*< 0.0001). There was a significant perch state and axis interaction in angular standard deviation (F_2, 98.4_ = 4.57, *P*< 0.0127). This was primarily due to higher variations in rotational movements about the craniocaudal (x) axis on the swaying perch. There was no significant effect of health state or challenge on variation in angular velocity. Angular velocity results are summarized in S1 Table Angular velocity measurements.

### Linear Acceleration

There were significant main effects of perch state (F_1, 79.5_ = 303, *P*< 0.0001) and axis (F_2, 107_ = 4.9, *P*< 0.009) on the number of peaks in linear acceleration (S3C Fig) with higher peaks (2.5 ± 0.1) on the swaying than on the static perch. (0.8± 0.1) and decreasing number of peaks about the mediolateral (y) axis (1.7 ± 0.1), craniocaudal (x) axis (1.64 ± 0.1) and dorsoventral (z) axis (1.58 ± 0.1) respectively. There was a significant interaction between axis and perch state on the number of peaks in linear acceleration (F_2, 107_ = 3.08, *P*< 0.05), which could be attributed to a higher intensity of linear dorsoventral movements on the swaying perch (x = 1.45±0.2, y = 1.49±0.2, z = 1.54±0.2), whereas on the static perch, linear movements were most intense in the mediolateral axis (x = 0.07±0.01, y = 0.08±0.01, z = 0.08±0.01). On the swaying perch, the number of peaks in linear acceleration were similar across all three axes (x = 2.51±0.1, y = 2.5±0.1, z = 2.45±0.1) whereas on the static perch, there was a higher number of peaks in the mediolateral axis (x = 0.76±0.01, y = 0.9±0.01, z = 0.72±0.01).

There were significant main effects of perch state (F_1, 83_ = 1665, *P*< 0.0001) and axis (F_2, 104_ = 40.6, *P*< 0.0001) on maximum linear acceleration with the highest corrective acceleration (7.2 ± 0.1 m s^-2^) occurring on the swaying perch in comparison to the static perch (1 ± 0.1 m s^-2^) and decreasing corrective accelerations about the dorsoventral (z) axis (4.3 ± 0.1 m s^-2^), versus the mediolateral and craniocaudal axes (each 4 ± 0.1 m s^-2^). There was a significant maximum linear acceleration interaction between axis and perch state (F_2, 104_ = 5.62, *P* = 0.0048), which could be attributed to the highest maximum corrective accelerations of movements occurring along the craniocaudal (x) axis on the swaying perch (7.12 ± 0.13 m s^-2^), whereas on the static perch, maximum intensity of linear acceleration movements were lowest along the craniocaudal axis (x = 0.80 ± 0.091 m s^-2^).There was a significant effect of environmental challenge (F_3, 83.3_ = 3.11, *P*< 0.03) on maximum linear acceleration (S3D Fig), where the highest corrective accelerations were performed in the non-environmental challenged (4.35 ± 0.15 m s^-2^) versus the challenged (4 ± 0.15 m s^-2^) condition (t_83.3_ = 1.98, *P* < 0.05).The maximum linear acceleration movements were not significantly different between healthy birds and birds with health problems.

There were significant main effects of perch state (F_1, 80.6_ = 387, *P*< 0.0001) and axis (F_2, 101_ = 15.5, *P*< 0.0001) on standard deviations of acceleration, with higher deviations occurring on the swaying perch (1.1 ± 0.04 m s^-2^) than on the static perch (0.3 ± 0.02 m s^-2^) and increasing variations about the craniocaudal (x) axis (0.66 ± 0.02 m s^-2^), the mediolateral (y) axis (0.678 ± 0.02 m s^-2^) and the dorsoventral (z) axis (0.68 ± 0.1 m s^-2^) respectively. There was a significant interaction between axis and perch state on standard deviation of acceleration (F_2, 101_ = 7.53, *P*< 0.001), which could be attributed to higher variations along the dorsoventral axis on the swaying perch (x = 1.09 ± 0.04 m s^-2^), whereas on the static perch, standard deviation was highest along the mediolateral axis (y = 0.27 ± 0.02 m s^-2^). Linear acceleration varied less for birds with higher body weights (F_1, 12.3_ = 10.24, *P*< 0.0074).

There was a significant interaction between axis and environmental challenge (F _6, 58.5_ = 2.31, *P*< 0.045), in which the variability of linear motion corrections along all three axes was the same in the masked challenge (0.71 ± 0.04 m s^-2^) whereas in all other test situations, variations in linear acceleration were different along the three axes (obstacle: x = 0.69 ± 0.05 m s^-2^, y = 0.70 ± 0.04 m s^-2^, z = 0.71 ± 0.05 m s^-2^; obstacle + mask: x = 0.58 ± 0.05, y = 0.6 ± 0.05, z = 0.61 ± 0.05; non-challenged: x = 0.68 ± 0.05, y = 0.71 ± 0.04, z = 0.71 ± 0.04). Linear acceleration results are summarized in S2 Table Linear acceleration measurements.

## Discussion

### Effects of Perch State

Laying hens rarely fell from perches. This result is in concordance with double-leg stance studies in humans, where only one incidence of loss of balance was recorded from 247 such trials [61]. Overall, hens in the present study only fell from the dynamic, swaying perch and not from the static perch. When visual information is altered (either from movement of the individual or the environment), individuals increase postural sway to correct actual or perceived center of mass displacements [17]. Human subjects standing on a platform moving antero-posteriorly in sinusoidal motions (similarly to the motion of the swaying perch in the present study) were found to have normal sway patterns with eyes open and irregular sway patterns with eyes closed, suggesting that vision does affect postural stability during dynamic perturbations [62]. However, this challenge imposed on chickens using a head mask did not result in significant differences on the incidence of falls between masked and unmasked birds This may suggest that masked birds were able to maintain balance by means other than visual input. Physically healthy people rely on 70% of somatosensory, 20% of vestibular and 10% of visual sensory input for balance. Nevertheless, on a swaying surface, they increase sensory weighing to vestibular and visual information as they decrease their dependence on somatosensory inputs for postural control [13].

Assuming the swaying perch is perceived as an unstable surface to the hens (as moving platforms are to humans), masked birds on the swaying perch would be expected to shift their reliance to the vestibular system for balance. The vestibular system in humans is essential for bipedal balance, while the vestibular system in birds is essential for flight. However, birds have a specialized organ involved in bipedal balance located within the lumbosacral vertebrae [30, 34, 63]. This lumbosacral organ consists of fluid filled canals between vertebrae on the dorsal wall of the lumbosacral vertebral canal that are thought to act like the semicircular canals of the vestibular system [30, 63]. Necker [41] observed that pigeons wearing a head mask were able to maintain balance on a rotating perch with the help of wing flaps. With lesions to the lumbosacral organ, however, masked birds had more difficulty maintaining balance. This mirrors the inability of humans with vestibular lesions to re-weigh sensory input for increased reliance on the vestibular system in order to balance on an unstable surface [64].

### Effects of Environmental Challenges

Birds did not intentionally jump from perches when their vision was disrupted by the head mask. In birds, vision is needed to estimate the timing and distance needed for a successful takeoff and landing [65]. Based on eye trajectories and varying flight paths in perch distance tests, Moinard et al. [66] suggested that to perform a successful jump between two static perches, laying hens likely gather visual cues (e.g. location, distance) from their intended landing point prior to takeoff. Thus, perhaps masked hens in the present study did not jump from the perch because it was not possible to plan their landing due to the lack of available visual cues. If masked birds were unable to willingly escape the swaying perch, there is an increased likelihood that these birds would fall off the swaying perch, but this was not the case in the present experiment. Perhaps this was influenced by the training period during which hens were rewarded with raisins after each successful perching event. During testing, hens may have been willing to remain on the perch despite the risk of falls from disrupted vision with the expectation of receiving a raisin reward afterwards.

Birds in environmentally challenging conditions (i.e. space limitations due to obstacles and disruption of vision via a head mask) reached the lowest maximum intensity for both linear and rotational movements overall (S3B and S3D Fig), and had the least variable linear movements. Masked birds demonstrated a crouched (as described by Taylor et al. [67]) and seemingly rigid/stiff posture on the swaying perch. One strategy employed by birds to negotiate a ground obstacle during bipedal locomotion is the assumption of a crouched posture [68–69], which minimizes the changes in the center of mass that would otherwise occur as the bird steps onto and over the obstacle. In the present experiment, since maximum linear acceleration, angular velocity, and linear standard deviation were lower in the masked (mask, obstacle + mask) challenges, it is possible that masked birds assumed a crouched posture as a means to increase stability in an unstable environment (swaying perch) when visual cues were unavailable. However, according to Daley and Biewener [68], a crouched posture requires increased muscle forces due to changes in leg posture and musculoskeletal gearing. Laying hens may also increase hind limb muscle activation as a balance strategy during dynamic perturbations or when visual input is unavailable, resulting in a crouched and rigid posture, which may limit postural control and increase the risk for falls and injuries, as was observed in humans by Park et al. [70].

Additionally, the presence of obstacles may limit the bird’s ability to perform proper wing flaps by disrupting the path of the wing. In humans, inhibiting arm movements as motor strategies for balance by fixing the arms to the side of the body when vision is disrupted increases instability [16]. On the swaying perch obstructed by obstacles, we noted that hens’ wings collided with the obstacles during wing flaps, which likely limited wing movements. In combination with crouched posture, limited wing use during balance perturbations of the swaying perch could explain why birds in the obstacle + mask condition had the least intense (S3B and S3D Fig) and variable movements compared to the other challenged birds. Impairment of vision by the mask may limit the amount of sensory input and obstruction of wing flap space (due to obstacles) which, in turn, may limit behavioural output that is necessary for proper balancing strategies. In such a case, crowded housing environments may place birds at a higher risk for falls when attempting to take-off or land within a tight space on a heavily occupied perch and possibly more so in dim lighting conditions where visual sensory input is reduced.

### Effects of Physical Health/State

In the non-challenged situation (when vision and no obstacles were provided), laying hens with physical impairments of the integument (foot pad and wing feather damage) left the aerial perch (by jumping or falling) earlier than healthy birds (S2 Fig). Research in wild birds demonstrated that coordination of force production between both legs and wings during take-off and landing contributes to controlled ascending and descending movements [71]. During take-off in wild birds, legs generate greater body accelerations compared with wings [72] while wings contribute proportionally more to changes in velocity during the last phase of landing [71]. Assuming that this could also be true for laying hens, an intact integument seems to be important for controlled and safe transitions. Soft tissue injuries, such as foot pad dermatitis/damage, can result in redness, heat under the skin, swelling, pain, and loss of function of the affected tissue. Behavioural responses to inflammation are beneficial in that they restrict movement and loading of appendages to allow time for healing and repair [73].The results of the present study seem to indicate that the act of perching in birds with foot pad dermatitis may be an unpleasant sensory and/or emotional experience associated with tissue damage.

While perching, plantar pressure/loading on the foot pad is an unavoidable component of the tendon locking mechanism leading to contraction of the flexor muscles acting on the toes [33]. This could have resulted in loading restriction of the feet by reducing the time spent perching. Perching time was reduced in birds with foot pad dermatitis in the non-challenged conditions (perch space was not limited, vision was provided) but not in the challenged conditions (S2 Fig). This was likely due to the birds' ability to jump using a wide-legged stance to bear less weight on the affected plantar surface. Plantar pain in humans has a destabilizing effect on bipedal stance, especially in the absence of vision, and even more so with disturbances to vestibular sensory input [74]. This not only suggests that unpleasant sensory experiences may impact postural control, but that birds affected by foot pad dermatitis may increase their reliance on visual cues to maintain/regain balance.

In wild birds, poor feather condition occurs immediately preceding and during moulting, which is a sensitive period where some species cannot be aerial without experiencing difficulties [75]. The action of jumping-off the aerial perch in birds with poor feather cover is perhaps due to poor wing feathering limiting the effectiveness of wing flapping as a balancing mechanism, thus leading to the decision to jump and reduce risk of falling. Having the opportunity to plan a flight path (no head mask), to lift the wings without touching obstacles (no obstacle), and to actively adjust posture to control landing conditions may also explain why birds with poor wing feathering did not remain on the perch under non-challenged conditions. This finding is in line with our hypothesis that birds with poor feather covering will spend less time perching.

Focusing on posture adjustments for balancing, we quantified the direction and intensity of a bird’s movement on the perch, with the gyroscope measuring a higher number of rotational movement corrections in birds with physical impairments than in healthy birds (S3A Fig). Rotational movements of the hens in the present study were least intense, least frequently performed and least variable about the dorsoventral axis. It is possible that hens have no need for rotations about the dorsoventral axis. These yaw motions are likely to be important for orienting the body to the left or right according to changes in path directions rather than for balance [76].

In humans, when a surface (e.g. the ground) shifts forward, the center of mass is displaced backwards in the opposite direction and the individual corrects this shift by re-adjusting the center of mass in the same direction (forward) as the surface shifts [77]. As with humans standing on a platform moving in sinusoidal back and forth motions [62], hens on the swaying perch moved more about the lateral axis (pitching forward and backward) vs the dorsoventral axis, especially since there was not a major yaw component to the perturbation of the perch's movement itself. They may also be able to deal with yaw more easily because having widespread feet gives them larger moments about which muscle forces can act to develop yaw torque. There may be self-stabilizing effects as well since a yaw perturbation will stretch their legs in opposite directions. If we assume each leg is a simple spring in this case, then there will be an automatic restorative force taking place. Another possible explanation for this result may be that hens in this study were restricted in this type of movement due to having both feet fixed/gripped to the perch. Although long axis rotation of the femur and thereby left/right rotation of the trunk is possible given the avian skeletal structure, this motion mainly occurs during locomotion during the swing phase of the feet [76], perhaps not during perching when the tendon locking mechanism is in place. In commercial housing systems, hens may encounter dynamic balance perturbation caused by pushing or crowding of birds not only in the front/back direction as was demonstrated by the swaying perch, but in other directions as well. Presumably, if dorsoventral rotation is limited during perching, an unexpected push to the cranial or caudal end of a perching hen by a conspecific may increase the risk of falls if the hen is unable to fully correct for this type of postural shift.

Lastly, birds with higher body weights had a lower number of falls from the perch and had less variable linear body movements. In birds, the majority of trunk weight lies in the pectoralis major (breast), the largest avian muscle which constitutes about 15 percent of body mass [39, 78]. In humans, obese and pregnant individuals both have an uneven distribution of body weight, mainly in the trunk region, which poses biomechanical constraints on the body that affect the ability to control posture [79]. If pectoral muscle weight were to affect body biomechanics just as excess weight does in humans, the heavier hens in this study would be expected to fall more from the perch and have higher variability in body movements. Instead, inertia provided by the pectoralis, located ventrally, appeared to assist with stability.

## Conclusion

Laying hens with and without physical health problems, in both challenged and unchallenged environments, successfully perched and remained off the ground. Environmental challenges and physical state had an effect on the ability to maintain balance, where laying hens used a combination of accelerations and rotations to stabilize themselves on a perch. In comparison to healthy birds, birds with physical health problems performed a higher number of rotational corrections to keep the body centered over the perch, thus indicating a more complex effort is required for them to maintain their balance. Less intense and less variable correction movements were performed under conditions with limited space and visual deprivation. This might suggest that balancing strategies are compromised in crowded housing environments and/or dim lighting conditions, putting birds at higher risk of falls and thereby injury. Injuries from falls are not only a welfare concern for the birds, but may also decrease egg production and quality due to pain and stress [42], which can have an economic impact on producers. Collectively, these results provide novel empirical support for the effectiveness of training on perch-balancing performance as well as highlight how overcrowding, low-light conditions, and poor physical health can reduce such performance. Overall, this study provides useful data as a starting point for future research and describes a method for objectively assessing the balance system of laying hens by quantifying correction movements.

## Supporting Information

S1 Dataset Data logger data in Matlab format files and R code files. (ZIP)

S1 Fig Orientation of linear (acceleration) and rotational (angular velocity) movements on (straight arrows) and about (curved arrows) the x (craniocaudal), y (mediolateral) and z (dorsoventral) axes relative to the bird. (TIF)

S2 Fig Latency to leave (falls or jumps) the perch (static or swaying) within the 30 second testing period (Foot = birds with foot pad dermatitis; Feather = birds with poor wing feathering; Keel = birds with keel damage; Healthy = birds without health conditions). Least square means ± SE are represented (*; P<0.05).
(TIF)

S3 Fig Effects of health condition, environmental challenges and perch state on angular velocity and linear acceleration. (TIF)

S1 Table Summary of values of the underlying statistic (F), degrees of freedom (df) and p-values comparing the significant effect of perch state, health status, axis and their significant interactions (*) on angular velocity measurements. (TIF)

S2 Table Summary of values of the underlying statistic (F), degrees of freedom (df) and p-values comparing the significant effect of perch state, health status, axis and their significant interactions (*) on linear acceleration measurements. (TIF)

S1 Video Swaying perch obstacle. (WMV)
