# Supplementary figures and images for: Physical Health Problems and Environmental Challenges Influence Balancing Behaviour in Laying Hens
Source: PLoS One. 2016 Apr 14;11(4):e0153477. doi: 10.1371/journal.pone.0153477 (PMC4831827; doi:10.1371/journal.pone.0153477)

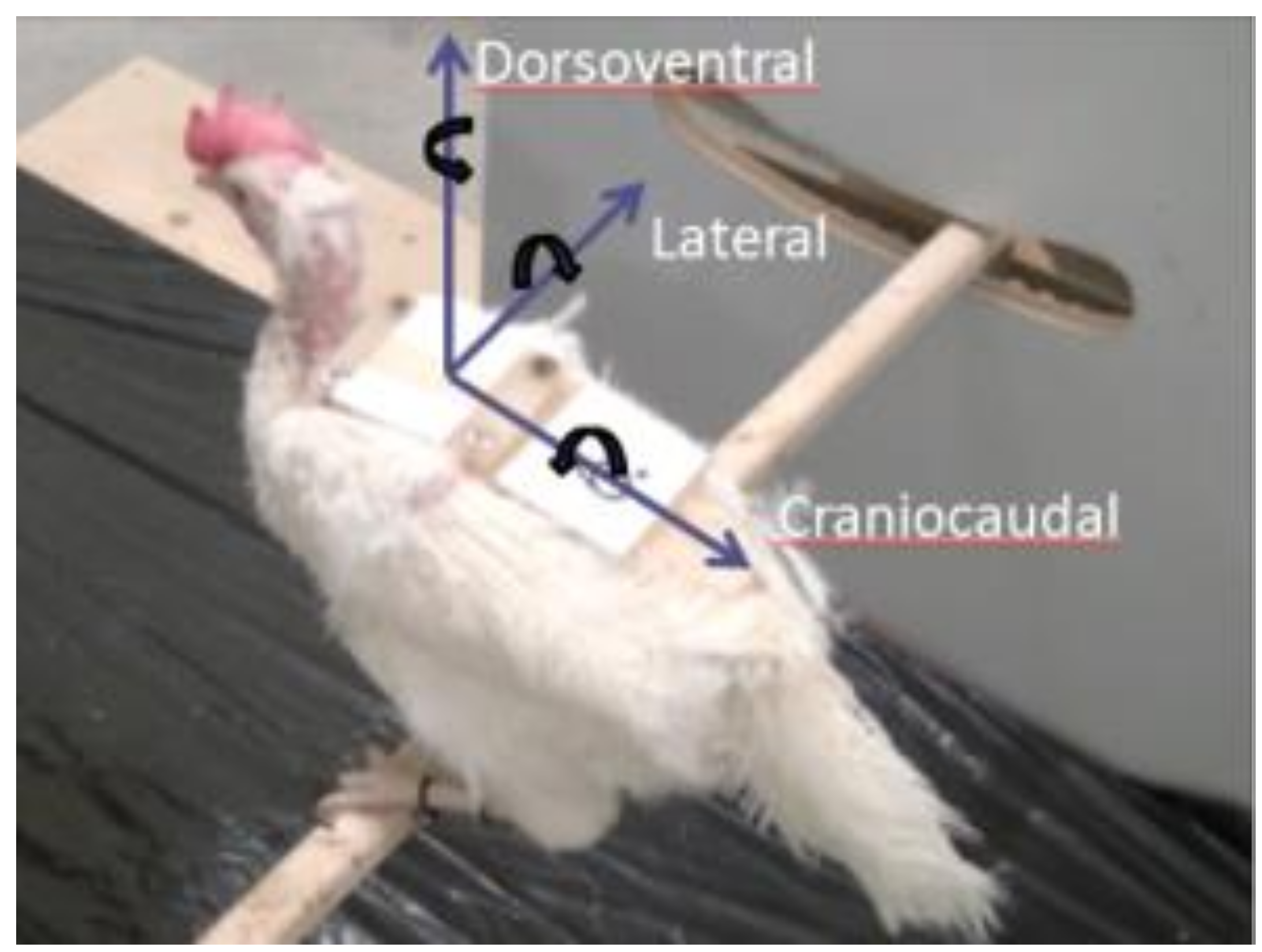

Supplement: S1 Fig — (TIF) [file pone.0153477.s002.tif]

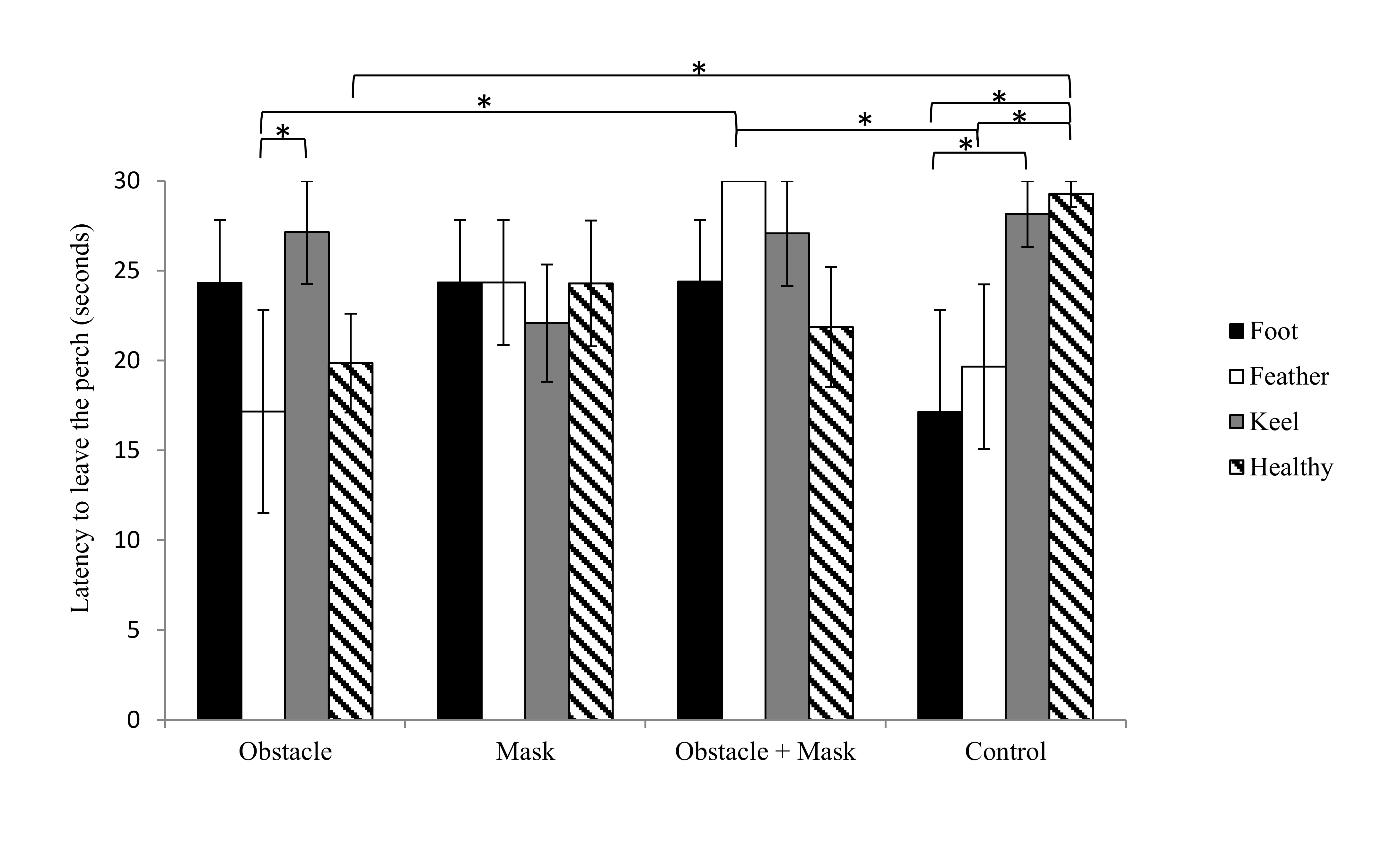

Supplement: S2 Fig — Least square means ± SE are represented (*; P<0.05). (TIF) [file pone.0153477.s003.tif]

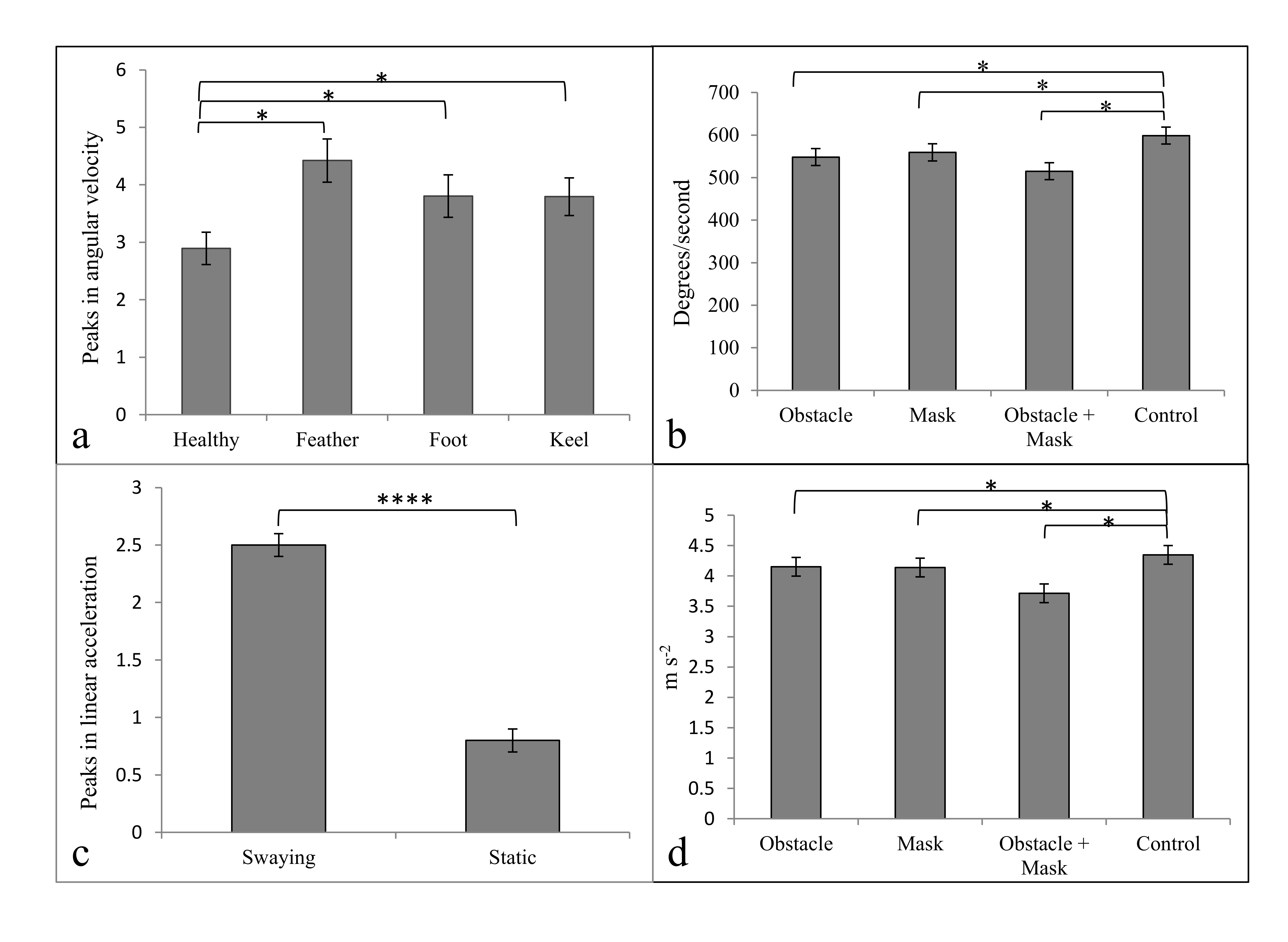

Supplement: S3 Fig — (TIF) [file pone.0153477.s004.tif]

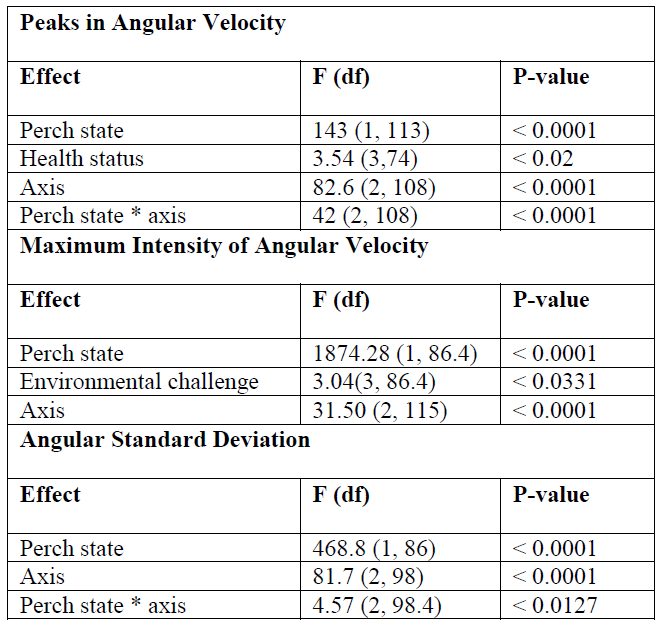

Supplement: S1 Table — (TIF) [file pone.0153477.s005.tif]

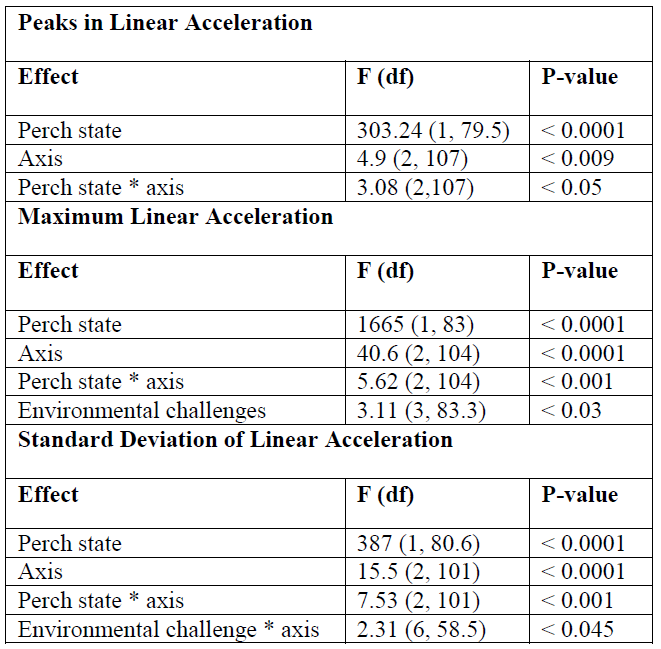

Supplement: S2 Table — (TIF) [file pone.0153477.s006.tif]
